# Prevalence and drug resistance pattern of *Listeria monocytogenes* among pregnant women in Tigray region, Northern Ethiopia: a cross-sectional study

**DOI:** 10.1186/s13104-019-4566-8

**Published:** 2019-08-23

**Authors:** Letemichael Negash Welekidan, Yemane Weldu Bahta, Mebrihit Gebremeskel Teklehaimanot, Getahun Kahsay Abay, Araya Gebreyesus Wasihun, Tsehaye Asmelash Dejene, Saravanan Muthupandian, Tadele Araya Mezgebo, Amlsha Kahsay Hagos

**Affiliations:** 1 Department of Medical Microbiology and Immunology, Division of Biomedical Sciences, College of Health Science, Mekelle University, P.O.Box:1871, Mekelle, Tigray Ethiopia; 2 Department of Obstetrics and Gynaecology, School of Medicine, College of Health Sciences, Mekelle University, P.O.Box:1871, Mekelle, Tigray Ethiopia; 3 College of Health Sciences, Environmental Microbiology, Aksum University, Axsum, Ethiopia

**Keywords:** *Listeria monocytogenes*, Pregnant women, Prevalence, Drug resistance, Tigray Region, Ethiopia

## Abstract

**Objective:**

The aim of this study was to determine the prevalence and antimicrobial susceptibility test of *Listeria monocytogenes* among pregnant women in Tigray region, Ethiopia.

**Results:**

The overall prevalence of *L. monocytogenes* among pregnant women was found to be (8.5%; 12/141). With regard to the socio-demographic characteristics, a high prevalence of *L. monocytogenes* was observed in the age group of 20–24 years (18.6%; 8/43), rural dwellers (10%; 3/30), secondary school (9.6%; 5/52), and housewives (11.4%;10/88). A high drug resistance rate was observed to penicillin G (66.7%), clindamycin (66.7%), amoxicillin (50%) and vancomycin (50%). However, isolates were relatively sensitive to ciprofloxacin (75%), erythromycin (75%), trimethoprim/sulphamethaxazole (66.7%) and chloramphenicol (60%).

## Introduction

Listeriosis caused by *Listeria monocytogenes,* is one of the important bacterial zoonotic infections worldwide [1]. This causes severe and life-threatening infection mainly in high-risk groups such as pregnant women, neonates, elderly and immunocompromised patients [2]. The fatality rate in high-risk groups can be up to 30% [3]. Studies revealed that listeriosis occurs 20 times more in pregnant women than the general people [4–7]. Teresa et al. [8], has also reported that 43% of the listeriosis cases conducted in 20 countries were related with pregnancy. In the United States listeriosis caused approximately 2500 serious illnesses and 500 deaths annually [9], and 17% of cases were associated with pregnancy [10].

Listeriosis in pregnant women is usually asymptomatic or with nonspecific clinical symptoms such as flu-like symptoms. However, it can cause abortion, preterm delivery, fetal death, or neonatal morbidity in the form of septicaemia, meningitis and encephalitis without being detected [8]. Approximately 20% of pregnancies complicated by listeriosis end in spontaneous abortion or stillbirth, and two-thirds of surviving infants develop clinical neonatal listeriosis such as pneumonia, sepsis, and it accounts for 20% of all cases of neonatal meningitis [7, 11–14]. The mortality rate for infants born alive approaches 20% and the frequency of abortion and stillbirth increases the overall mortality rate to more than 50% [8]. Previous studies reported drug resistance for *L. monocytogenes* like penicillin G, cefotaxim, gentamicin [15–18].

Foods which are commonly contaminated by *Listeria* such as ready-to eat foods, uncooked meats, fish, uncooked vegetables, unpasteurized milk and milk products should not be eaten by pregnant women, and are suggested to undergo screening for the bacteria [19]. Despite the potential threat to human health posed by this pathogen, there is scarcity of data on listeriosis among pregnant women in Ethiopia, particularly in Tigray there is no published data on pregnant women. Therefore, the aim of this study was to determine the prevalence and antimicrobial susceptibility test of *L. monocytogenes* among pregnant women attending antenatal care of Mekelle Hospital and Ayder comprehensive specialized hospital.

## Main text

### Materials and methods

#### Study area and design

A hospital based, cross-sectional study was conducted from February to May 2016 at Mekelle hospital and Ayder comprehensive specialized hospital, Northern Ethiopia. Mekelle is located at 783 km north of Addis Ababa, the capital of Ethiopia. Mekelle, which is the capital city of Tigray regional state, has a total population of 307,305 [20]. A total of 141 pregnant women who attended at the hospitals antenatal care with a flue like signs and symptoms were included in the study.

#### Eligibility criteria

Pregnant women having flue like signs and symptoms such as fever, backache, headache, vomiting/diarrhea, muscle pains and sore throat was included in the study.

#### Sample size and sampling technique

A total of 141 pregnant women who fulfil the eligibility criteria were consecutively recruited from February to May 2016.

#### Dependent variables

Prevalence of *L. monocytogenes* and antimicrobial susceptibility test.

#### Independent variables

Socio-demographic variables.

#### Data and specimen collection

Clinical and demographic data were collected using structured questionnaire. After written informed consent obtained from study participant, 5 ml blood sample was collected aseptically, transferred into a sterile 0.6% tryptose soy broth (Oxoid, UK) in a screw caped test tube, and transported to Ayder microbiology laboratory within 1 h.

#### Culture and identification

After overnight incubation on Tryptic soy broth (Oxoid, UK) plus 0.6% yeast extract enrichment broth. The suspected growth were sub-cultured to palkam agar media and listeria selective agar (Oxoid, UK), and incubated at 35 °C for 24 h. The green shiny colonies with diffuse black shadow around them on palkam agar and yellow small colonies on listeria selective agar were suspected to be *Listeria*. Suspected colonies were further identified using gram stain, catalase test, motility test, beta haemolysis on blood agar, CAMP test, fermentation of sugars (xylose, rhamnose, mannitol and methyl d-mannopyranoside), oxidase test and methyl red-voges proskauer (MR-VP) tests.

#### Antimicrobial susceptibility testing

A standard Kirby–Bauer disk diffusion method was used [21]. Inoculum suspension was prepared using sterile saline to obtain turbidity comparable to 0.5 McFarland standards and Sterile cotton swab was dipped, rotated across the wall of the tube to avoid excess fluid and was evenly inoculated on Muller-Hinton agar (Oxoid, UK) and the antibiotic discs were placed on Muller-Hinton agar plates.

The following antibiotics were tested: penicillin G (10U), trimethoprim/sulphamethaxazole (25 μg), ciprofloxacin (5 μg), amoxicillin (20 μg), erythromycin (15 μg), clindamycin (2 μg) and vancomycin (30 μg) (Oxoid, UK).

#### Quality assurance

Standard operational procedures were followed and *L. monocytogenes* (ATCC 7644) was used as the reference strain.

#### Data analysis

Data were analyzed using SPSS version 20. Descriptive statistics and binary logistic regression were employed. Binary logistic regression was used to show the association of each variable with the dependent variable. P-value < 0.05 with 95% confidence interval was considered statistically significant.

### Results

In the present study, 141 pregnant women enrolled. The mean age of participants was 26.23 (+ 5.42 SD). Of the total, 84 (59.6%), 111 (78.7%), 78 (55.3%), 88 (62.4%) and 67 (47.5) participants were in the age range 20–29, urban dwellers, attended secondary school and above, house wives and had fever/headache, respectively (Table 1).

**Table 1:** Association of socio-demographic characteristics of pregnant women (n = 141) with *L. monocytogenes* in Mekelle Hospital and Ayder Comprehensive Specialized Hospital, Tigray, Ethiopia [Feb–May, 2016]

Variable	Frequency	Percent (%)	*L. monocytogenes*		P-value	COR (95% CI)
			Positive	Negative		
Age (years)						
15–24	58	41.1	8 (13.8)	50 (86.2)		1
25–34	68	48.2	3 (4.4)	65 (95.6)	0.077	3.47 (0.88–13.74)
35–44	15	10.6	1 (6.7)	14 (93.3)	0.465	2.24 (0.26–19.46)
Residence						
Urban	111	78.7	9 (8.1)	102 (91.9)		1
Rural	30	21.3	3 (10)	27 (90)	0.742	0.79 (0.20–3.13)
Educational status						
Unable to read and write	14	9.9	1 (7.1)	13 (92.9)	0.95	1.08 (0.09–13.1)
Read and write	13	9.2	1 (7.7)	12 (92.3)	1	1 (0.08–12.16)
Elementary school	36	25.5	3 (8.3)	33 (91.7)	0.927	0.92 (0.14–5.92)
Secondary school	52	36.9	5 (9.6)	47 (90.4)	0.78	0.78 (0.14–4.34)
College level	26	18.4	2 (7.7)	24 (92.3)		1
Occupation						
Government employee	13	9.2	1 (7.7)	12 (92.3)		1
Merchant	20	13.5	1 (5)	19 (95)	0.753	1.58 (0.09–27.77)
Daily worker	21	14.9	1 (4.8)	20 (95.2)	0.727	1.67 (0.1–29.18)
House wife	87	62.4	9 (10.3)	78 (89.7)	0.767	0.72 (0.08–6.22)

#### Prevalence of *Listeria monocytogenes*

The overall prevalence of *L. monocytogenes* among pregnant women was 8.5% (12/141). A high prevalence of *L. monocytogenes* was observed in the age group of 15–24 (13.8%; 8/58), rural dwellers (10%; 3/30), those who attended secondary school (9.6%; 5/52), and housewives (10.3%; 9/87) respectively, but not statistically significant (Table 1).

#### Clinical data of the pregnant women

Study participants with fever (8; 11.9%), headache (6; 9%), muscle pain (3; 7.9%), vomiting (2; 11.1%) and diarrhea (2; 28.6%) had *L. monocytogenes* infection.

#### Risk factors for *Listeria monocytogenes* infection

Pregnant women who had a frequent habit of consuming unpasteurized milk were 2.9 times at risk for *L. monocytogenes* infection, but it was not statistically significant (COR = 2.9 (10.57–0.79), P = 0.108). On the other hand, consumption of fish, meat and uncooked vegetables were not found as risk factor for *L. monocytogenes* infection (Table 2).

**Table 2:** Association of possible risk factors for *L. monocytogenes* infection among pregnant women attending antenatal care clinics in Mekelle Hospital and Ayder Comprehensive Specialized Hospital, Tigray, Ethiopia [Feb–May, 2016]

Variable	Total	*L. monocytogenes*		P-value	COR (95% CI)
		Positive	Negative		
Gestational age					
1st trimester	65	6 (9.2)	59 (90.8)	0.709	0.73 (0.14–3.85)
2nd trimester	47	4 (8.5)	43 (91.5)	0.8	0.8 (0.14–4.65)
3rd trimester	29	2 (6.9)	27 (93.1)		1
Frequently feeding habit					
Packed meat					
Yes	1	0	1 (100)	1.0	1.515E8 (0.00)
No	140	12 (8.6)	128 (91.4)		1
Fish					
Yes	6	0	6 (100)	0.999	1.576EB (0.00)
No	135	12 (8.9)	123 (91.1)		1
Uncooked vegetables					
Yes	136	12 (8.2)	124 (91.8)	0.999	0.00 (0.00)
No	5	0	5 (100)		1
Unpasteurized milk					
Yes	118	8 (6.8)	110 (93.2)	0.108	2.9 (0.79–10.57)
No	23	4 (17.4)	19 (82.6)		1

#### Antimicrobial susceptibility test of *Listeria monocytogenes*

**Fig. 1:**
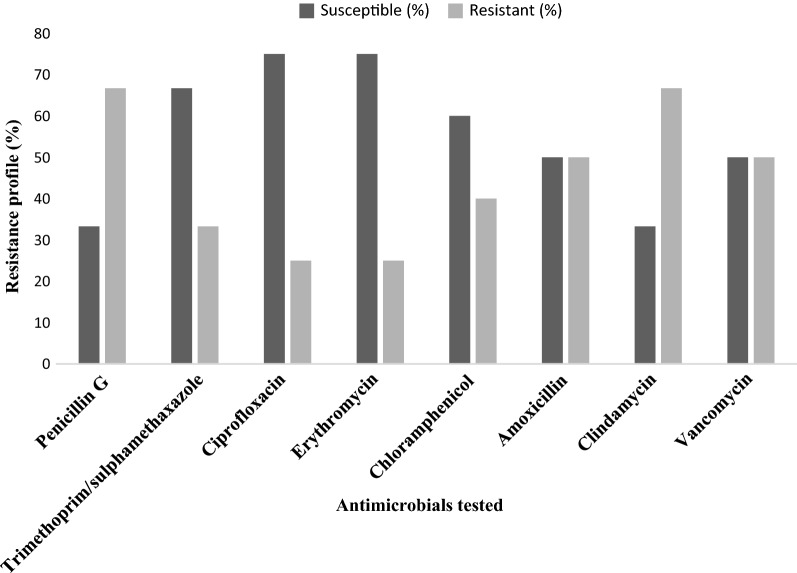
Antimicrobial susceptibility test of *L. monocytogenes* isolates among pregnant women attending antenatal care clinics in Mekelle Hospital and Ayder Comprehensive Specialized Hospital, Tigray, Ethiopia [Feb–May, 2016]

Drug resistance to antibiotics like penicillin G (66.7%), clindamycin (66.7%), amoxicillin (50%), and vancomycin (50%) were found to be high, whereas relatively low rate of drug resistance were observed to the antibiotics chloramphenicol (40%), trimethoprim/sulphamethaxazole (33.3%), ciprofloxacin (25%) and erythromycin (25%) (Fig. 1).

### Discussion

In this study, a considerable prevalence of *L. monocytogenes* was found among pregnant women. This may cause mild infection to the pregnant mothers but it can be devastating to the fetus that can result to preterm delivery, stillbirth, fetal death, or a serious neonatal morbidity like septicemia, pneumonia, meningitis and encephalitis. The prevalence of this study (8.5%; 12/141) is comparable with research findings (8.9%) done in HIV/AIDS patients in Nigeria [22] and a case report (9.3%; 11/118) in British [23]. However, it is higher than the findings from United States done among general population (4.8%; 7/147) [10] and New England in a case series and review on pregnant women (5.0%; 11/222) [13]. However, it is lower than findings from systemic review and meta-analysis (20.7%) [24] and a case report from Ireland in 2007 (42.9%; 9/21) [25]. The possible explanations for the differences could be due to difference in study groups, sample size, awareness and geographic location.

The prevalence of *L. monocytogenes* among pregnant women found to be higher in the age group 20–24 years, and house wives. The possible reasons could be due to lack of awareness on the source of infection, risk of infection and preventive measures. In this study, the prevalence of *L. monocytogenes* was higher in the first trimester gestational period. Early gestational Listeria infection of the fetuses have lesser chance of recovery [13] than later gestations, and mostly the outcome ends up with spontaneous abortion or stillbirth [26–28]. Studies reported that the outcome of early gestational listeriosis was spontaneous abortion (10–20%), preterm delivery (50%), intrauterine fetal death (11%) [28], fetal distress (34%), and meconium staining of the amniotic fluid (75%) [13, 28]. Even though, preterm birth is common [29], infants born at the earliest gestations have the highest mortality rate [28]. However, listeriosis mostly reported in the third trimester [30], so far, cases at earlier gestational period have been diagnosed [31, 32]. The possible reason for rarity of the incidence of listeriosis during earlier gestational period could be due to unfrequently culturing of products of conception/fetal tissue in cases of early fetal losses [33]. The other possible reason for the higher prevalence of *L. monocytogenes* in the first trimester gestational period could be due to other predisposing factors to the pregnant women. A report from case series and review mentioned that some pregnant women were taking immunosuppressive drugs for heart transplantation secondary to cardiomyopathy, and some pregnant women developed gestational diabetes during the first trimester of pregnancy [13].

Pregnant women who had the habit of consumption of unpasteurized milk (6.8%) were infected with *L. monocytogenes.* This implies pregnant women should have to avoid foods known to be at increased risk of contamination with *L. monocytogenes.* Out of 142 reported cases of listeriosis in epidemics associated with contaminated Mexican-style cheese prepared from unpasteurized milk, 65.5% occurred in pregnant women [34]. Although, unpasteurized milk is a common risk factor for acquisition of listeriosis, pasteurized milk associated outbreak in Massachusetts reported that 14% (7/49) of cases were pregnancy associated [35].

In this study, high drug resistances were observed on penicillin G (66.7%), clindamycin (66.7%), amoxicillin (50%), and vancomycin (50%). Therefore, development of drug resistance for the advisable drugs may face a great challenge in the treatment of listeriosis that can be threatening for the outcome of pregnancy. However, unlike to this study, all *L. monocytogenes* isolated from spontaneous abortion of humans in Iran were sensitive to trimethoprim and erythromycin (100% each) and relatively higher sensitivity to chloramphenicol (88%) and ciprofloxacin (66.67%) [15]. Besides, all *L. monocytogenes* isolated from ready to eat foods in Poland were sensitive to ciprofloxacin, chloramphenicol and trimethoprim/sulfamethoxazole [36]. Moreover, isolates from Chinese food were highly susceptible to ciprofloxacin (90.5%) and higher susceptibility to trimethoprim/sulfamethoxazole (57.1%) were reported [37]. Comparing to the current study, higher drug resistance were reported from a study done on HIV/AIDS patients in Nigeria to amoxicillin (100%), trimethoprim-sulfamethoxazole (79.3%), chloramphenicol (62.1%), erythromycin (48.3%) and ciprofloxacin (31%) [22], ciprofloxacin (37%) resistance for isolates from Poultry in Georgia [38], and penicillin G (77.77) resistance of isolates from spontaneous abortion of humans in Iran [15]. However, lower resistance were observed for isolates from foods and human samples in Germany to erythromycin (1.9%) and ciprofloxacin (9.7%) [39], in China on Chinese food to clindamycin (52.4%) [37], and chloramphenicol (11.11%) for isolates from spontaneous abortion of humans in Iran [15]. The extensive utilization of antibiotics in human medicine, veterinary medicine and agriculture has attributed for the increasing emergence of drug resistant bacteria, including strains of *Listeria* spp. [40].

## Conclusion and recommendations

High prevalence of *L. monocytogenes* and rate of drug resistance were found among pregnant women. Therefore, pregnant women should be aware of source of infection, preventive measures, morbidity, and mortality rates by focusing on the risks like abortion, preterm delivery and stillbirth. Active screening for *L. monocytogenes* infection and early treatment is also required during pregnancy to prevent the possible complications to the mother and the fetus.

## Limitations

- This study did not look for the status of HIV in the study participants.
- Did not study the strains circulating in the region.

## Data Availability

The data of this study is available with the corresponding author up on request.
